# Cross sectional association between cognitive frailty and disability among community-dwelling older adults: Focus on the role of social factors

**DOI:** 10.3389/fpubh.2023.1048103

**Published:** 2023-02-09

**Authors:** Kyungwon Choi, Young Ko

**Affiliations:** ^1^Department of Nursing, Korea National University of Transportation, Chungju, Chungbuk, Republic of Korea; ^2^College of Nursing, Gachon University, Incheon, Republic of Korea

**Keywords:** cognition, frailty, social relationships, disability, elderly

## Abstract

**Background:**

This study aimed to investigate the prevalence of cognitive frailty and the influence of social factors on the association between different levels of cognitive frailty and disability.

**Methods:**

A nationally representative survey of non-institutionalized community-dwelling older adults in Korea was used. A total, 9,894 older adults were included in the analysis. We assessed the effects of social factors using social activities, social contacts, living arrangements, emotional support, and satisfaction with friends and neighbors.

**Results:**

The prevalence of cognitive frailty was 1.6%, which was consistent with other population-based studies. Hierarchical logistic analysis demonstrated that the association between different levels of cognitive frailty and disability was attenuated when social participation, social contact, and satisfaction with friends and community were included in the model, and the magnitude of these effects differed across the levels of cognitive frailty.

**Discussion:**

Considering the influence of social factors, interventions to enhance social relationships can help slow down the progression of cognitive frailty to disability.

## 1. Introduction

In the context of worldwide aging, frailty is considered a public health concern because it is directly related to adverse health outcomes such as disability, hospitalization, institutionalization, falls, and mortality. A broad consensus on the definition of frailty is that it is a multidimensional geriatric syndrome that manifests a critical reduction in the functional and physiological reserves of multiple organic systems (1). From a multidimensional perspective, psychosocial factors (2, 3), cognitive function (2–4), and environmental factors are considered in the context of frailty.

Studies have demonstrated that physical factors and cognition are crucial elements in predicting the risk of mortality (5, 6), and that frailty and cognitive impairment may happen at the early stages of disability and dementia (7–9). There is still insufficient to fully understand the complexities of the combined conditions of frailty and cognitive impairment, although significant relationship between frailty and cognitive impairment has been established in the literature (4, 10). In recognition of the importance of both physical and cognitive functions, the International Academy on Nutrition and Aging and the International Association of Gerontology and Geriatrics defined cognitive frailty as a heterogeneous clinical manifestation characterized by the presence of both frailty and cognitive impairment in the absence of a clinical diagnosis of dementia (11). Subsequent studies revealed that people with cognitive frailty had a high risk of limitations on instrumental activities of daily living (IADLs), functional disability, poor quality of life, falls, hospitalization, death, and incident dementia (2–4). Therefore, appropriate support and timely interventions aimed at preventing or reducing the process of cognitive frailty and adverse health outcomes should be developed.

It is well-known that social inclusion positively influences health outcomes in older adults, such as physical frailty, cognitive impairment, and maintaining functional ability. Accordingly, high participation in social activity and frequent social contact have been associated with delayed progression to cognitive impairment (12, 13) and physical frailty (14, 15) among older adults. Similarly, the likelihood of functional decline is the highest in older adults with a lack of social contact (16, 17) or social participation (18, 19). A lack of satisfaction with social support (20, 21) or lack of good relations with relatives (17) was also associated with greater difficulties in activities of daily living (ADLs) and IADLs in older adults. However, few studies have explored whether social factors influence the association between cognitive frailty and adverse health outcomes, thus contributing to the slowing down of the transition from cognitive frailty to disability. Moreover, while most studies have not investigated the association between social factors and adverse health outcomes across levels of frailty, some have found that the effects of social factors on frailty varied across levels of physical frailty (22, 23).

Based on these findings, we aimed to investigate the prevalence of cognitive frailty in Korea and examine whether social factors influence the relationship between cognitive frailty and disability, and how the relationship differs across the levels of cognitive frailty. We hypothesized that social factors would impact the association between different levels of frailty and disability, and the effects of these factors would vary according to the level of cognitive frailty.

## 2. Materials and methods

### 2.1. Study design

This was a cross-sectional study with secondary data from the 2017 National Survey of Older Koreans (NSOK) (24). The protocol for the secondary analysis was approved by the Investigational Review Board of the university with which the researchers were affiliated (IRB No. 1044396-202107-HR-150-01).

### 2.2. Study setting and participants

NSOK has been conducted every 3 years since 1988. NOSK 2017 took place through in-person interviews in 934 survey areas from June 12 to August 28, 2017. The target population was non-institutionalized older adults aged 65 years or older and living in the community. A sample of older adults was selected using a stratified two-stage cluster sample design. The number of samples was calculated based on the 2010 population and housing census data (24). A total of 10,299 older adults participated in the 2017 survey; however, in this study, data from only 9,894 participants were used, after excluding those who had missing data on the main variables (*n* = 405) or were diagnosed with dementia (*n* = 149).

### 2.3. Measurement

#### 2.3.1. Disability

Disability was measured using the Korean instrumental activities of daily living (K-IDL) scale (25). Disability was defined as requiring partial or full assistance for at least one activity.

#### 2.3.2. Cognitive frailty

Cognitive frailty was operationally defined as a score ≥ 3 in the physical frailty criteria with cognitive impairment. Physical frailty was assessed using five items: fatigue, resistance, ambulation, illness, and weight loss (26). The five items were assessed and evaluated as follows: (i) Fatigue: 0 points for “no” and 1 point for “yes” to the question, “Have you lost a lot of activity or motivation these days?” (ii) Resistance: for the question “How difficult is it to climb 10 steps without any break?” “not difficult at all” or “slightly difficult” were scored 0 points, and “very difficult” or “not at all” were scored 1 point; (iii) Ambulation: for the question “How difficult is it to walk about one lap (400 m) on the playground?” “not at all difficult” or “slightly difficult” were scored 0 points, and “very difficult” or “not at all” were scored 1 point; (iv) Illness: 0 points if the number of diseases diagnosed by a doctor (hypertension, diabetes mellitus, cancer, chronic bronchitis/emphysema, angina pectoris/myocardial infarction, other heart disease, asthma, arthritis, cerebrovascular accident (cerebral infarction or stroke), chronic kidney disease) was 0–3, and 1 point for four or more diseases; and (v) Loss of weight: in the case of those who responded that they lost or gained more than 5 kg in weight despite not intentionally controlling their weight within 6 months, those who were underweight were scored 1 point, and the rest were scored 0 point. Based on these five criteria, those who fell into none of the above were defined as robust, those with one or two criteria were pre-frail, and those who fulfilled more than three criteria were defined as physically frail (27).

Cognitive function was assessed with the Mini-Mental State Examination for Dementia Screening (MMSE-DS) (28). The MMSE-DS consists of 19 items, and the total score is calculated by summing all items. Normal and cognitive impairments were classified using the criterion score based on gender, age, and educational level (28).

The participants were divided into four groups based on their levels of physical frailty and cognitive function. Participants without physical frailty and with normal cognitive function were classified as the “Robust” group. If the participants had no physical frailty but had cognitive impairment, they were part of the “Cognitively impaired” group. If the participants had physical frailty but no cognitive impairment, they were assigned to the “Physical frailty” group. Participants with physical frailty and cognitive impairment were classified into the “Cognitive frailty” group.

#### 2.3.3. Social factors

Social factors included structural and functional variables in this study. We included living arrangements, frequency of participation in social activities, and the number of close persons as structural aspects. We included emotional support and satisfaction with friends and the community as the functional aspects. Living arrangements were classified as follows: living alone, no spouse, living with others, and living with a spouse and/or others. The frequency of social activity participation per week was calculated by summing the frequency of seven social activities per week: club, social club, political and social group, volunteer activity, religious activity, senior citizen's center, and welfare center for seniors. The frequency of participation in social activities per week was classified as less than once per week, once per week, 2–3 times per week, and four or more times per week. Social contact was assessed using the question, “How many relatives, friends, neighbors, and acquaintances, including brothers and sisters, do you have close to (with whom you can confide in your mind)?” and classified as no social contact at all, 1–2 people, and three or more people.

The emotional support received from the participants' children, parents, or spouses was measured. Emotional support was measured based on the extent of assistance provided through counseling. An item was scored based on a Likert scale, where 1 was “extremely unlikely,” 2 was “unlikely,” 3 was “likely,” and 4 was “extremely likely.” The participant was assigned 0 when they did not have children, parents, or spouses. Higher scores indicated a higher level of support. Life satisfaction with friends and community was measured using the question “To what extent are you satisfied with relationships with friends and society?” The response options were: 1, very satisfied; 2, satisfied; 3, average; 4, not satisfied; and 5, not satisfied at all. “Very satisfied” and “satisfied” were classified as “satisfied,” and “average,” “dissatisfied” and “very dissatisfied” were classified as “dissatisfied.”

#### 2.3.4. Covariates

We considered covariate variables such as age, gender, educational attainment, equivalent family income, and subjective health status as possible factors influencing disability. Average household incomes were calculated by dividing the total household income by the square root of the number of household members. Equivalent family income was classified into quartile groups based on the distribution (lowest 25, 25–50, 50–75, and highest 25%). Subjective health status was measured on a 5-point scale in response to the question “How do you feel about your general health?” (1, very unhealthy; 2, unhealthy; 3, average; 4, healthy; and 5, very healthy).

### 2.4. Data collection

For the 2017 NSOK, a trained surveyor visited the participants' homes (dwellings) and conducted the survey directly with trusted respondents using a structured questionnaire. For this study, we were provided with data without personal identification information from the Korea Institute for Health and Social Affairs.

### 2.5. Data analysis

To analyze the general characteristics, social factors of participants and the prevalence of disability, descriptive statistics were used. The χ^2^-test or ANOVA with Scheffe test were performed to compare the differences among four groups. Hierarchical logistic analysis was used to identify the influence of cognitive frailty on disability and the role of social factors in the association between the levels of cognitive frailty and disability. Disability was the dependent variable in these analyses. In Model 1, covariates (general characteristics and health-related characteristics) and cognitive frailty groups were entered. In Model 2–6, each of social factors was added to Model 1 and in model 7, covariates, social factors, and cognitive frailty groups were included. After adding social factors, the percentage of change in the odds ratio (OR) [explained fraction = [(OR model B-OR model A)/(OR model B-1)] × 100] before [Model Before (B)] and after adding [Model After (A)] was presented to identify the degree of contribution of social factors. This is useful for measuring the direct and indirect contributions of social factors to the association between levels of cognitive frailty and disability (29). Statistical analyses were carried out with the SPSS software (version 26.0 for Windows; SPSS, Inc., Chicago, IL, USA).

## 3. Results

### 3.1. Characteristics of the participants and disability

Table 1 presents the general characteristics, social factors of the participants, and prevalence of disability. Regarding the classification of the cognitive frailty group, 79.3% were in the robust group, 14.8% in the cognitive impairment group, 4.2% in the physical frailty group, and 1.7% in the cognitive frailty group. Older persons in the cognitive frailty group had the lowest level of social participation, social support, emotional support, and showed least satisfaction with friends and community. Those in the physical frailty group showed higher levels of social support and satisfaction with friends and community than those in the cognitively impaired group. The percentage of older adults participating social activities for 2 and more days per week were higher in the physical frailty group than in the cognitively impaired group. The overall rate of disability was 22.2%. Among the four groups, the disability rate was highest in the cognitive frailty group. Significant differences in the prevalence of disability were observed in sex, age, educational attainment, equivalent family income, and subjective health status (*p* < 0.001). In addition, the prevalence of disability showed a significant difference in all social relation factors, such as living arrangement, social participation, social support, emotional support, and satisfaction with friends and community (*p* < 0.001).

**Table 1:** General characteristics and social factors according to cognitive frailty groups and prevalence of disability (*N* = 9,894).

**Characteristics**	**Category**	**All**	**Robust group^a^**	**COI + robust group^b^**	**Non-COI + frail group^c^**	**CoI + frail group^d^**		**Prevalence of disability**
		***n* (%) or M ±SD**	***n* (%) or M ±SD**	***n* (%) or M ±SD**	***n* (%) or M ±SD**	***n* (%) or M ±SD**	***p***	
Total		9,894 (100.0)	7,927 (79.3)	1,355 (14.8)	453 (4.2)	159 (1.7)		
Prevalence of disability		22.2	17.5	24.8				
Gender	Male	3,990 (42.8)	3,082 (41.0)	756 (58.7)	93 (22.2)	59 (36.2)	< 0.001	13.9^†^
	Female	5,904 (57.2)	4,845 (59.0)	599 (41.3)	360 (77.8)	100 (63.8)		28.4
Age (years)	65–74	5,253 (58.6)	4,331 (60.3)	770 (61.6)	111 (28.6)	41 (26.8)	< 0.001	11.8^†^
	≥75 years	4,641 (41.4)	3,596 (39.7)	585 (38.4)	342 (71.4)	118 (73.2)		36.9
Educational attainment	(0–22)	7.21 ± 4.59 (a,b > c,d)	7.36 ± 4.65	7.31 ± 4.05	4.84 ± 4.51	5.57 ± 4.39	< 0.001	
	Elementary school	6,092 (57.7)	4,767 (55.9)	852 (60.8)	361 (76.6)	112 (72.4)	< 0.001	30.4^†^
	Middle	1,581 (17.1)	1,264 (17.2)	248 (18.6)	42 (10.3)	27 (15.9)		12.6
	More than high	2,221 (25.2)	1,896 (26.9)	255 (20.6)	50 (13.1)	20 (11.7)		9.8
Equivalent family income (1,000 KRW)^a^	≤ 854.2	2,457 (22.9)	1,921 (22.2)	304 (20.8)	173 (38.6)	59 (34.3)	< 0.001	32.6^†^
	854.3–1,282.7	2,477 (23.2)	1,976 (23.2)	337 (22.6)	123 (25.5)	41 (24.5)		24.0
	1,282.8–2,057.7	2,480 (25.7)	1,956 (25.1)	396 (30.5)	93 (20.4)	35 (23.5)		19.8
	≥2,061.3	2,480 (28.2)	2,074 (29.5)	318 (26.1)	64 (15.5)	24 (17.7)		14.3
Subjective health status	(1–5)	2.96 ± 0.98 (a,b > c,d)	3.05 ± 0.95	2.91 ± 0.97	1.89 ± 0.70	1.88 ± 0.75	< 0.001	
	Unhealthy	4,040 (39.1)	2,883 (34.9)	584 (42.1)	401 (89.0)	142 (88.1)	< 0.001	38.0^†^
	Average	2,320 (23.5)	1,984 (24.9)	296 (22.6)	30 (6.5)	10 (6.5)		18.2
	Healthy	3,554 (37.4)	3,060 (40.2)	475 (35.3)	22 (4.5)	7 (5.4)		8.2
Living arrangement	Living alone	2,502 (23.9)	2,021 (24.1)	279 (18.8)	163 (37.0)	39 (27.2)	< 0.001	31.8^†^
	Elderly couple	4,895 (48.9)	3,960 (49.3)	279 (51.8)	163 (36.1)	39 (37.2)		15.9
	Living with others	2,497 (27.1)	3,960 (26.5)	703 (29.3)	166 (27.0)	66 (35.7)		25.1
Social participation (days per week)	< 1 days	2,754 (28.5)	1,946 (25.0)	512 (38.8)	196 (43.4)	100 (66.6)	< 0.001	30.9^†^
	1	2,422 (25.9)	1,981 (26.4)	331 (26.3)	86 (19.5)	24 (16.5)		16.6
	2–3	2,746 (27.3)	2,293 (28.7)	325 (22.3)	104 (23.9)	24 (11.2)		20.3
	≥4	1,972 (18.3)	1,707 (19.9)	187 (12.6)	67 (13.2)	11 (5.7)		19.2
Social support (person)		2.29 ± 2.67 (a,b > c,d)	2.40 ± 2.67	2.10 ± 2.78	1.43 ± 2.27	0.95 ± 1.48	< 0.001	
	None	3,184 (31.6)	2,354 (28.9)	528 (38.8)	211 (46.7)	91 (57.2)	< 0.001	32.6^†^
	1–2	3,277 (32.9)	2,647 (33.3)	421 (30.6)	161 (34.5)	48 (30.3)		22.0
	≥3	3,433 (35.6)	2,926 (37.9)	406 (30.7)	81 (18.8)	20 (12.5)		13.1
Emotional support	(0–4)	2.73 ± 0.71 (a > d)	2.75 ± 0.70	2.70 ± 0.69	2.66 ± 0.82	2.56 ± 0.99	< 0.001	
	Less (0–2.9)	3,596 (38.0)	2,790 (36.8)	533 (41.5)	202 (44.4)	71 (46.0)	< 0.001	22.5
	More (3–4)	6,298 (62.0)	5,137 (63.2)	822 (58.5)	251 (55.6)	88 (54.0)		22.0
Satisfaction with friends and community	(1–5)	2.48 ± 0.79 (a > c > b > d)	3.60 ± 0.75	3.35 ± 0.82	3.01 ± 0.96	2.71 ± 0.80	< 0.001	
	Unsatisfied	3,793 (35.1)	2,724 (49.5)	651 (64.3)	287 (83.6)	131 (39.3)	< 0.001	31.2^†^
	Satisfied	6,101 (64.9)	5,203 (50.5)	704 (35.7)	166 (16.4)	28 (60.7)		22.2

^†^ < 0.01 for prevalence differences among different levels of each variable.

### 3.2. Cognitive frailty and disability: Role of social factors

Table 2 shows the influence of cognitive frailty and social factors on disability. In Model 1, after adjusting for the covariates, the probability that the cognitive frailty group was disabled was 1.92 (1.65–2.24) times higher than that of the robust group. Compared to the robust group, the probability of having a disability was 7.01(5.41–9.09) times higher in the physical frailty group, and 15.36 (9.53–24.76) times higher in the cognitive frailty group than the robust group. In addition, the probability of having a disability was higher in those who were female, older than 74 years, with a low educational level, and low subjective health status.

**Table 2:** Odds ratio and 95% confidence intervals (CI) for disability among Korean older persons aged 65 or older.

**Characteristics**	**Comparison group (reference)**	**Model 1**	**Model 2**	**Model 3**	**Model 4**	**Model 5**	**Model 6**	**Model 7**
		**OR (95% CI)**	**OR (95% CI)**	**OR (95% CI)**	**OR (95% CI)**	**OR (95% CI)**	**OR (95% CI)**	**OR (95% CI)**
Gender	Female (male)	1.48 (1.30–1.68)^**^	1.39 (1.22–1.59)^**^	1.57 (1.38–1.78)^**^	1.56 (1.37–1.77)^**^	1.47 (1.30–1.67)^**^	1.52 (1.34–1.73)^**^	1.54 (1.34–1.76)^**^
Age	≥75 years (65–75 years)	2.78 (2.49–3.12)^**^	2.71 (2.42–3.04)^**^	2.83 (2.52–3.17)^**^	2.71 (2.42–3.04)^**^	2.79 (2.49–3.12)^**^	2.78 (2.48–3.11)^**^	2.68 (2.39–3.01)^**^
Equivalent family Income	Q1 (Q4)	1.07 (0.90–1.27)	1.17 (0.98–1.41)	1.06 (0.90–1.25)	1.04 (0.88–1.22)	1.05 (0.89–1.24)	1.03 (0.87–1.21)	1.09 (0.91–1.32)
	Q2 (Q4)	1.09 (0.92–1.30)	1.04 (0.87–1.24)	0.95 (0.80–1.12)	0.94 (0.80–1.11)	0.94 (0.80–1.11)	0.93 (0.79–1.10)	1.00 (0.84–1.20)
	Q3 (Q4)	1.08 (0.90–1.29)	1.02 (0.86–1.21)	0.97 (0.82–1.12)	0.96 (0.81–1.14)	0.96 (0.81–1.13)	0.95 (0.80–1.13)	1.01 (0.85–1.20)
Educational attainment		0.85 (0.84–0.86)^**^	0.85 (0.84–0.86)^**^	0.85 (0.84–0.86)^**^	0.85 (0.84–0.86)^**^	0.85 (0.84–0.86)^**^	0.85 (0.84–0.86)^**^	0.86 (0.84–0.87)^**^
Subjective health status		0.55 (0.51–0.58)^**^	0.55 (0.52–0.58)^**^	0.56 (0.52–0.59)^**^	0.56 (0.53–0.60)^**^	0.55 (0.52–0.58)^**^	0.57 (0.53–0.60)^**^	0.58 (0.54–0.61)^**^
Cognitive frail group	Cognitive impairment group (robust)	1.92 (1.65–2.24)^**^	1.91 (1.64–2.22)^**^	1.85 (1.58–2.15)^**^	1.87 (1.61–2.18)^**^	1.92 (1.65–2.24)^**^	1.83 (1.57–2.13)^**^	1.76 (1.51–2.05)^**^
	Physical frailty group (robust)	7.01 (5.41–9.09)^**^	7.00 (5.40–9.07)^**^	6.65 (5.13–8.63)^**^	6.86 (5.29–8.90)^**^	6.99 (5.40–9.06)^**^	6.36 (4.90–8.25)^**^	6.27 (4.82–8.15)^**^
	Cognitive frailty group (robust)	15.35 (9.53–24.76)^**^	15.13 (9.39–24.40)^**^	13.80 (8.53–22.33)^**^	14.51 (8.97–23.46)^**^	15.26 (9.47–24.60)^**^	13.01 (8.04–21.06)^**^	12.32 (7.57–20.04)^**^
Living arrangement	Living alone (living with spouse)		1.18 (1.02–1.35)^*^					1.21 (1.05–1.40)^**^
	Living with others (living with spouse)		1.33 (1.15–1.54)^**^					1.26 (1.09–1.47)^**^
Social participation (days per week)	1 (< 1)			0.72 (0.62–0.84)^**^				0.79 (0.68–0.93)^**^
	2–3 (< 1)			0.66 (0.57–0.77)^**^				0.76 (0.66–0.89)^**^
	≥4 (< 1)			0.65 (0.56–0.77)^**^				0.77 (0.65–0.92)^**^
Social support (closed persons)	1–2 (none)				0.68 (0.59–0.77)^**^			0.71 (0.63–0.81)^**^
	≥3 (none)				0.63 (0.55–0.73)^**^			0.73 (0.63–0.84)^**^
Emotional support						0.96 (0.89–1.04)		1.03 (0.95–1.11)
Satisfaction for friends and community							0.77 (0.72–0.82)^**^	0.84 (0.78–0.91)^**^
Percentage change^†^	Cognitive impairment group (robust)		1.1%	7.6%	5.4%	0.0%	9.8%	17.4%
	Physical frailty group (robust)		0.2%	6.0%	2.5%	0.3%	10.8%	12.3%
	Cognitive frailty group (robust)		1.5%	10.8%	5.9%	0.6%	16.3%	21.1%

^†^Percentage change in ORs for disability compared with the reference model was calculated using the following formula: i.e., Model 1; [(OR (Model 1) – OR (Model 2–8) / (OR (Model 1) – 1) × 100]].

^*^p < 0.05,

^**^p < 0.001.

OR, Odds ratio; CI, Confidence interval.

In Models 2–6, each social relationship factor was sequentially added to Model 1 to explore the effect of each factor on the odds of cognitive frailty for disability. Living arrangement, social support, social participation, and satisfaction with friends and community accounted for 1.5, 5.9, 10.8, and 16.3% of the effect of cognitive frailty on disability, respectively. All the Odds ratios for social factors except emotional support in each model were statistically significant, indicating that poor social relationships increased the risk of disability in older adults.

In Model 7, after adding all social factors to Model 1, the probability that the cognitively impaired group had a disability was 1.76 (1.51–2.05) times higher than that in the robust group. It was decreased by 17.4% compared to the Odds ratio in the cognitively impaired group in Model 1. Compared with robust group, the Odds ratio of disability in the physical frailty group was 6.27 (4.82–8.15) times higher in Model 7. It was decreased by 12.3% compared to the Odds ratio in the physical frailty group in Model 1. The Odds ratio in the cognitive frailty group was also 12.32 (7.57–20.04) times higher than those of the robust group in Model 7. It was decreased by 21.1% compared to the Odds ratio in the cognitive frailty group in Model 1.

## 4. Discussion

This study investigated the prevalence of cognitive frailty in Korea and examined the impact of social factors on the association between the levels of cognitive frailty and disability. The prevalence rate of cognitive frailty (1.6%) in this study was in line with other population-based studies, which ranged from 1.0 to 4.4 in community-based settings (2, 30–33). In contrast, some studies in community-based settings have shown higher prevalence rates of cognitive frailty. In a study of 1,751 older persons aged 65 years and older from the Manitoba Study of Health and Aging (MSHA) (34) and in Xie's study of 1,586 Chinese older adults aged 75 years and older (35), the prevalence rates of CF were 12.0 and 7.2%, respectively. The higher prevalence rates in those studies may be explained by the higher mean age of the sample population compared to other studies (77.5 and 81.4, respectively).

The results of this study clearly showed that individuals with comorbidities of physical frailty and cognitive impairment have a higher risk of disability than older adults with either physical frailty or cognitive impairment alone, as well as healthy older adults. This is in line with previous studies which suggested that since both physical frailty and cognitive impairment may be related to an increased risk of adverse health outcomes, older adults with their co-occurrence are more likely to be at a particularly high risk (3, 36).

Specifically, we found that the association between different levels of cognitive frailty and disability was attenuated when social relationship variables were included in the model, and the magnitude of the attenuation was largest in cognitively frail adults, followed by older adults with cognitive impairment only. That is, our results reveal that the beneficial effects of social factors on the association between different levels of cognitive frailty and disability were greater for older adults with cognitive impairment than for those with physical frailty. This is in line with previous studies reporting that outcomes of the social dimension (22) and effects of intervention (37) vary depending on the level of frailty. These studies demonstrated that older adults with a transitional status—neither a progressive high frailty group nor stable as the least frail group—were more influenced by modifiable variables, including social support. Likewise, in this study, older adults in the cognitive frailty group were not only physically frail but cognitively impaired, which means that they have a higher risk of dementia (36) or disability (2, 32) than older adults with either physical frailty or cognitive impairment only. Our findings suggest that health care providers should develop and provide the continuous interventions to enhance social relationships for older adults with cognitive frailty as well as older adults with cognitive impairment or physical frailty only.

As expected, all associations between social factors and different levels of cognitive frailty and disability were in the same direction. However, the strength of the association and the relative importance of the social factors were different. Among social factors, the change in Odds of different levels of cognitive frailty in relation to disability was the largest when satisfaction with friends and the community was included in the model; the second largest change in OR was with the addition of social participation in the model. According to the theory of socioemotional selectivity proposed by Carstensen (38), social relationships change with age, which is the result of a selection process that develops over life, and older adults primarily maintain social relations to maximize emotional closeness. Thus, subjective satisfaction with relationships including friends, community may be more influential on the health outcomes of older adults than the structural aspects of social relationships, which is supported by previous studies (39, 40). Participation in social activities and social contacts was most consistently associated with preservation of global cognitive function (13, 41–43) and prevention or slowing down of the process of physical frailty (44) across all study types. The theory of “use it or lose it” (45) suggests that the brain can be considered a muscle; thus, social activities may stimulate the brain and contribute to the preservation of cognitive function. In addition, participation in social activities may decrease the risk of loneliness and depression, which are important risk factors for physical frailty (46). It also increases physical activity, which can improve the maintenance of physical function (9, 47, 48). According to the stress-buffering hypothesis, social activities may benefit health outcomes through their buffering effect on stress levels (49). Participation in social activities may provide opportunities for interacting with others in one's social network and increase the availability of various types of social support (46). These processes may influence cognitive functioning by reducing stress and lowering the levels of stress hormones (46).

Although it was not the primary aim of the current study, the results showed several previously observed associations between social factors and disability. As expected, social participation and social contact had a negative relationship with the risk of disability in older adults. Interestingly, however, the strength of the association did not show large differences across the frequencies of social participation, which was the same across the number of close persons, when all social factors were included in the model. Many studies have demonstrated that a higher level of social participation is associated with a higher level of functional status among older adults (19). A prospective study by Ide et al. (19) reported a dose-response relationship between social participation and functional decline; thus, a higher level of social participation is associated with a higher level of functional status among older adults. An exception is the study by Yokobayashi et al. (50), who reported that more frequent contact with friends was not associated with improved glycemic control, suggesting an optimal frequency of meeting friends (1–4 times per month) that may contribute to better glycemic control. Our result is in line with the study by Yokobayashi et al. (50), in that a higher frequency of social participation did not guarantee relatively stronger relationships with higher levels of health outcomes. Our findings suggest that the participation in social activities and social contact even once a week may play a role in slow down the progression of disability for older adults. However, we could not determine the causal relationship between the social factors and the different levels of cognitive frailty because the design of the present study was cross-sectional. Further prospective studies are needed to examine the strength of causal relationship.

There are more caveats in the interpretation of these results in addition to cross-sectional design of this study. We used the FRAIL and MMSE-K scales to assess level of frailty and cognitive function. Although the instruments for measuring frailty and cognitive function were consistent with previous research on older populations (2, 26, 36, 51, 52), we cannot rule out the possibility that the prevalence of cognitive frailty could vary if different measures are used. Moreover, we did not consider a broad spectrum of social activities or specific cognitive domains. Future studies should use multiple instruments to measure various aspects of the two concepts, such as capturing frequencies and types of social participation and specific cognitive ability, such as working memory, attention, verbal fluency, and processing speed. Despite these limitations, our study used a nationally representative sample weighted by census estimates, thereby increasing the generalizability of these findings. This study also shows the importance of social participation, social contacts, and satisfaction with friends and community in delaying the progression of cognitive frailty to disability, although each social variable has different effects across different levels of cognitive frailty and presents important implications for public health policy.

## Data availability statement

The original contributions presented in the study are included in the article/supplementary material, further inquiries can be directed to the corresponding author.

## Ethics statement

The studies involving human participants were reviewed and approved by Gachon University (IRB No. 1044396-202107-HR-150-01). The patients/participants provided their written informed consent to participate in this study.

## Author contributions

KC and YK conceived and designed the study, analyzed the data, and wrote the first draft. Both authors contributed to revisions of the manuscript, critical discussion, read, and agreed to the published version of the manuscript.
